# Association of cardiac and renal function with extreme N-terminal fragment Pro-B-type natriuretic peptide levels in elderly patients

**DOI:** 10.1186/1471-2261-12-57

**Published:** 2012-07-26

**Authors:** Hua Cui, Guoliang Huo, Lin Liu, Li Fan, Ping Ye, Jian Cao, Yongyi Bai, Fan Wang, Yixin Hu

**Affiliations:** 1 Second Geriatric Cardiology Division, Chinese PLA General Hospital, No 28 Fuxing Road, Beijing, 100853, P. R. China

**Keywords:** NT pro-BNP, Factors, Elderly, Impaired renal function

## Abstract

**Background:**

The data are inconsistent regarding whether extreme N-terminal fragment pro-B-type natriuretic peptide (NT pro-BNP) levels are associated with impaired renal function. Furthermore, the relationship between extreme NT pro-BNP levels and cardiac and renal function in elderly patients has not been reported. The aim of the present study was to examine a hypothesis that extreme NT pro-BNP levels may be associated with impaired cardiac and renal function in elderly patients.

**Methods:**

We retrospectively analyzed the data of demographic, clinical, and echocardiographic features on 152 consecutive elderly patients aged more than 80 years old (average age, 83.65 ± 3.58 years) with NT pro-BNP levels ≥ 3000 pg/ml. The participants were divided into two categories according to their NT pro-BNP levels: (1) 3000–10000 pg/mL and (2) >10000 pg /mL.

**Results:**

The number of patients with impaired renal function (P = 0.019) and the mortality (P < 0.001) in the period of inpatient was higher in the group with NT pro-BNP > 10000 pg /mL. The levels of serum creatinine and creatine kinase MB (CK-MB) in the group of NT pro-BNP > 10000 pg / mL were higher than those in the group of NT pro-BNP = 3000-10000 pg/mL (P = 0.001 and P = 0.023, respectively). Furthermore, no significant difference in the distribution by NYHA class in different NT pro-BNP levels was observed. Multiple linear regression analyses demonstrated that with NT pro-BNP levels as the dependent variable, NT pro-BNP levels were positively correlated with CK-MB (β = 0.182, P = 0.024) and creatinine levels (β = 0.281, P = 0.001). The area under the receiver-operating characteristic (ROC) curve of NT pro-BNP levels and clinical diagnosis of impaired renal function was 0.596 and reached significant difference (95%CI:0.503-0.688, P = 0.044).

**Conclusion:**

These data suggest that the extreme elevation of NT pro-BNP levels (≥3000 pg/ml) is mainly determined by impaired renal function in elderly patients above 80 years. Extreme NT pro-BNP levels may be useful for assessing the severity of impaired renal function.

## Background

B-type natriuretic peptide (BNP; 77–108 amino acids) and its N-terminal (1–76 amino acids) counterpart, NT-proBNP, are cardiac biomarkers that have been established for the assessment of left ventricular dysfunction and congestive heart failure. Respecting NT pro-BNP has a longer half-life than BNP, measurements of circulating levels of NT pro-BNP have been prior recommended in the diagnosis and prognosis of patients with symptoms of left ventricular dysfunction [1-3]. In addition, NT pro-BNP testing is also important in the diagnostic and prognostic evaluation of patients with Chronic kidney disease (CKD) [4]. However, Law et al. [5] revealed that extreme values of BNP were not associated with kidney dysfunction or the presence of HF, cardiomyopathy. Interestingly, Guglin et al. [6] demonstrated extremely high BNP levels (4000–20,000 pg/mL) were correlated with renal dysfunction measured by serum creatinine levels.

CKD is regarded as an important problem in elderly patients, which is an independent risk factor for cardiovascular disease. The prevalence of CKD rises rapidly in individuals older than 60 years of age [7]. It is well documented that adults lose about 1 mL/min/y of the glomerular filtration rate (GFR) from the age of 40 to 45 years. Given the increased incidence of impaired renal function and NT pro-BNP levels in elderly patients [8], we hypothesized that extreme NT pro-BNP levels may be associated with impaired renal function in elderly patients. Furthermore, the relationship between extreme NT pro-BNP levels and cardiac and renal function in elderly patients has not been reported. The aim of the present study was to examine the hypothesis that extreme NT pro-BNP levels may be associated with impaired cardiac and renal function in elderly patients.

## Methods

### Ethical approval of the study protocol

This study complied with the Declaration of Helsinki. It was approved by the Scientific and Ethics Review Board of the Department of Geriatrics, Chinese PLA General Hospital (Beijing, PR China). All patients provided written informed consent to be included in the study.

### Participants

We screened for elderly patients ≥ 80 years, hospitalized between April 2010 and January 2011, whose NT pro-BNP values were ≥ 3000 pg/mL regardless of diagnosis. According to the NT pro-BNP levels, the patients were divided into two categories: 3000–10000 pg/mL and > 10000 pg/mL. The cutoff of NT pro-BNP level was chosen arbitrarily.

NT pro-BNP was determined by chemiluminescence immunoassay, ADVIA Centaur™ system (Roche Inc.), and was expressed as pg/ml. The normal value for the NT pro-BNP test was <150 pg/mL. From the values >3000 pg/mL measured during the same hospital admission, we calculated the average number. Laboratory indicators, such as C-reactive protein (CRP), albumin, creatine kinase MB (CK-MB) and creatinine levels,which may be correlated with the NT pro-BNP level, were also collected on the same day when NT pro-BNP exceeded 3000 pg/mL.

We reviewed medical records of all patients enrolled, including history and physical examination findings, progress notes and the data of instrumental and laboratory diagnostic tests. From the clinical records available, we made the diagnosis of heart failure (HF) and estimated functional class by the New York Heart Association (NYHA).

Left ventricular ejection fraction (LVEF) was calculated by means of echocardiographic study using Simpson’s rule. Other parameters of structural and functional of heart, such as left atrial diameter, right atrial diameter, interventricular septum, posterior wall, left ventricular end systolic diameter (LVESD) and left ventricular end diastolic diameter (LVEDD), fractional shortening (FS) were also reviewed from electronic medical records closed to the day when NT pro-BNP exceeded 3000 pg/mL. Serum creatinine levels were recorded on the same day that NT pro-BNP exceeded 3000 pg/mL. Impaired renal function was defined as a serum creatinine level > 1.5 mg/dL [9].

### Statistical analysis

Results are presented as mean ± SD, as median and interquartile ranges, or as percentages and numbers for categorical data. Normality and homogeneity of variances were tested for all variables. NT pro-BNP was natural logarithmically transformed to normalize their distributions. The Student’s t-test or Mann–Whitney U two-sample tests (if the distribution was not normal) were used to compare the continuous variables between the two groups. Categorical data and proportions were analyzed using the Mann–Whitney U or kruskal Wallis H test where appropriate. Variables potentially associated with the plasma concentration of NT pro-BNP in all subjects were analyzed by linear regression analyses. The relationship between NT pro-BNP and clinical diagnosis of HF or impaired renal function was tested with the area under the receiver-operating characteristic (ROC) curve. P < 0.05 was considered statistically significant. All analyses were performed using SPSS for Windows version 16.0 (SPSS, Chicago, IL, USA).

## Results

### Patient characteristics

Demographics are provided in Table 1. One hundred and fifty-two consecutive patients (91 men and 61 women) with an age ranging from 80 to 94 (mean age, 83.65 ± 3.58 yr) were enrolled and underwent statistical analysis. Among them, the NT pro-BNP levels of 108 patients (66 men) were between 3000 pg/mL and 10000 pg/mL (group A), 44 (25 men) ≥10000 pg/mL (group B). Of the 152 participants, 16 patients admitted with acute myocardial infarction, 69 with HF or HF exacerbation, and 24 patients died during their hospitalization. Among 83 patients without HF symptoms, the main reason of being hospitalized, including infections (35 patients); brain damage such as stroke and Parkinson’s disease (11 patients); cancer (25 patients); and miscellaneous conditions (remaining 12 patients).

**Table 1:** Baseline characteristics of the enrolled patients with elevated NT pro-BNP levels

	**NT pro-BNP 3000-10000 pg/mL ( group A, n = 108)**	**NT pro-BNP ≥10000 pg/mL (group B, n = 44)**	***P***
Age (years)	83.57 ± 3.33	83.84 ± 4.18	0.679
Male, n (%)	66 (61.1)	25 (56.8)	0.625
BMI (kg/m2)	22.64 ± 3.79	21.50 ± 3.33	0.084
SBP(mm Hg)	126.69 ± 20.21	128.39 ± 23.19	0.653
DBP (mm Hg)	68.25 ± 12.37	66.32 ± 16.54	0.487
Underlying commodities disease, n (%)			
Coronary artery disease	89(82.4)	37(84.1)	0.803
Atrial fibrillation	43(39.8)	15(84.1)	0.447
Diabetes mellitus	37(34.3)	19(43.2)	0.303
Hypertension	81(75.0)	36(81.8)	0.367
COPD	13(12.0)	4(99.1)	0.602
Respiratory failure	13(12.0)	7(15.9)	0.523
Cancer	29(26.9)	10(22.7)	0.860
Liver disease	10(9.3)	5(11.4)	0.694
MODS	6(5.6)	1(2.3)	0.383
Impaired renal function	39(36.1)	25(56.8)	0.019
Infection	57(52.8)	28(63.6)	0.223
Acute myocardial infarction	7(6.5)	4(9.1)	0.951
Death	9(8.3)	15(34.1)	<0.001
Echocardiographic parameters			
LVEF (%)	54.88 ± 10.44	53.41 ± 9.98	0.427
Left atrial diameter (mm)	39.39 ± 6.43	38.07 ± 5.34	0.231
Right atrial diameter (mm)	37.81 ± 6.85	36.39 ± 5.22	0.216
Interventricular septum (mm)	10.69 ± 1.57	10.80 ± 1.75	0.729
Posterior wall (mm)	10.06 ± 1.28	10.14 ± 1.75	0.753
LV diastolic dimension (mm)	49.98 ± 6.51	50.55 ± 5.42	0.613
LV systolic dimension (mm)	35.67 ± 6.41	35.93 ± 5.74	0.265
Fractional shortening (%)	29.81 ± 5.72	29.20 ± 4.79	0.297
Laboratory data			
Creatinine (mg/dL)	1.24(0.92-1.82)	1.39(0.91-4.57)	0.001
CK-MB (ng/dL)	2.5(0.90-368)	3.63(1.72-5.77)	0.023
Serum albumin(g/dL)	33.44 ± 5.11	32.83 ± 6.76	0.540
C-reactive protein (mg/dL)	3.07(0.86-7.58)	3.44(1.025-8.6)	0.375
Medication, n (%)			
ACEIs or ARBs	7 (6.5)	20 (45.5)	0.803
β-blockers	16(36.4)	48(44.4)	0.362

NT pro-BNP, N-terminal fragment pro-B-type natriuretic peptide;NS, not significant; BMI, body mass index; SBP, Systolic blood pressure; DBP, diastolic blood pressure ;HF, heart failure; LV, left ventricular; MODS, multiple organ dysfunction syndrome; CK-MB, MB isoenzyme of creatine kinase; COPD, chronic obstructive pulmonary disease. Data were shown as mean ± SD, percentages and median values (quartile 1, quartile 3) for different parameters.

There were no significant differences between the group A and the group B, when comparing age; having atrial fibrillation, diabetes mellitus, hypertension, cancer, chronic obstructive pulmonary disease or liver diseases; echocardiographic parameters; and medications. More patients with impaired renal function (P = 0.019) and higher mortality (P < 0.001) in the period of hospitalization were found in the group B when compared to group A. The levels of serum creatinine and the CK-MB in the group B were higher than that in the group A (P = 0.001, P = 0.023 respectively), but no difference trend appeared in serum albumin and C-reactive protein between the two groups.

No significant difference in the distribution by NYHA class in different NT-pro BNP levels was observed between the group A and B presented in Table 2.

**Table 2:** Distribution patients by NYHA class in different NT pro-BNP Levels

	**NT pro-BNP 3000–10000 (pg/mL, %) ( group A, n = 108)**	**NT pro-BNP ≥10000 (pg/mL, %) ( group B, n = 44)**	***P***
NYHAI	23 (21)	7 (16)	NS
NYHAII	36 (34)	13 (30)	NS
NYHAIII	31 (38)	15 (34)	NS
NYHAIV	18 (17)	9 (20)	NS

NYHA, New York Heart Association class; NT pro-BNP, N-terminal fragment pro-B-type natriuretic peptide.

### Correlation and ROC analyses

**Figure 1:**
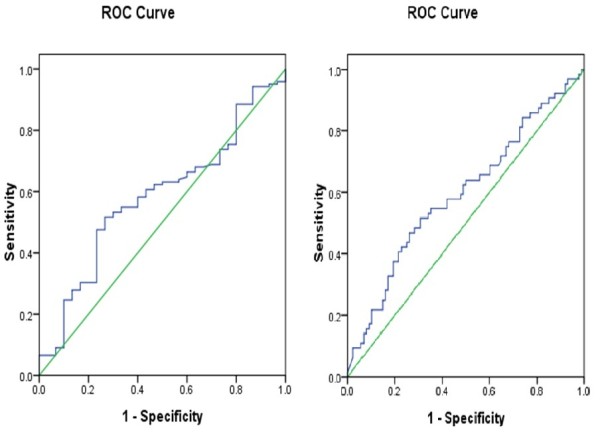
**The ROC curve of NT pro-BNP levels and clinical diagnosis of HF (Figure A) and impaired renal function (Figure B).** AUC = area under the curve; CI = confidence interval; ROC = receiver-operating characteristic.

Correlation analyses showed that NT pro-BNP levels were positively correlated with CK-MB (r = 0.162, P = 0.050) and creatinine (r = 0.237, P = 0.003). Multiple linear regression analyses demonstrated that with NT-pro BNP levels as the dependent variable, NT pro-BNP levels were positively correlated with CK-MB (β = 0.182, P = 0.024) and creatinine (β = 0.281, P = 0.001)(Table 3). The area under the ROC curve of NT pro-BNP levels and clinical diagnosis of HF was 0.582 and was not significant (95%CI:0.474-0.690, p = 0.164) (Figure 1). However, the area under the ROC curve of NT pro-BNP levels and clinical diagnosis of impaired renal function was 0.596 and reached significant difference (95%CI:0.503-0.688, p = 0.044) (Figure 1).

**Table 3:** Correlation between clinical data, echocardiographic parameters, laboratory data and NT pro-BNP levels

	**Univariate**		**Multivariate**	
	***r***	***P***	***β***	***P***
Age	−0.017	0.832	-	NS
BMI	−0.134	0.099	−0.151	0.058
Systolic blood pressure	−0.029	0.726	-	NS
Diastolic blood pressure	−0.122	0.134	-	NS
Echocardiographic parameters				
LVEF	−0.085	0.300	-	NS
Left atrial diameter	−0.031	0.701	-	NS
Right atrial diameter	−0.093	0.254	-	NS
Interventricular septum	−0.078	0.341	-	NS
Posterior wall	−0.022	0.787	-	NS
LV diastolic dimension	0.054	0.512	-	NS
LV systolic dimension	0.028	0.730	-	NS
Fractional shortening	0.012	0.883		NS
Laboratory data				
Creatinine	0.237	0.003	0.281	0.001
CK-MB	0.162	0.050	0.182	0.024
Serum albumin,	−0.078	0.337	-	NS
C-reactive protein	0.131	0.341	-	NS

NT pro-BNP, N-terminal fragment pro-B-type natriuretic peptide; NS, not significant; BMI, body mass index; SBP, systolic blood pressure; DBP, diastolic blood pressure; HF, heart failure; LV, left ventricular; CK-MB, MB isoenzyme of creatine kinase.

## Discussion

In the present study, we found extreme NT pro-BNP levels were positively correlated with creatinine levels and the evaluation of impaired renal function in elderly patients. Elevated NT pro-BNP levels are common in patients with CKD, as it showed in the presence of coronary artery disease (CAD) and left ventricular hypertrophy (LVH). Nitta and colleagues [10] reported that the mean serum NT pro-BNP concentration was higher in haemodialysis patients than in normal healthy subjects. Yang et al. [11] demonstrated that BNP levels of patients with CKD showed a positive correlation with creatinine levels, and a high concentration of BNP can be seen in patients with stage 5 CKD without heart failure. After analyzing 277 patients with CKD, Prnjavorac et al. [12] found that NT pro-BNP increased at the beginning of water overload in patients with CKD. However, Law et al. [5] demonstrated that BNP levels were not correlated with the presence of renal dysfunction in 113 patients with BNP value >3000 pg/mL. The major reason for this inconsistency maybe that patients with an age ranging from 21 to 102 were enrolled in Law’s study , which may lead to different result regarding BNP levels determined by age. Moreover, our results are similar to the study by Guglin et al. [6], who also found high BNP levels (4000–20,000 pg/mL) were associated with kidney dysfunction. Although the data are inconsistent, our results suggest in elderly patients, the extreme NT pro-BNP may be more useful for the assessment of the severity of impaired renal function. Certainly, further studies are still needed to ascertain the relationship between extreme BNP levels and impaired renal function in large sample size.

Previous evidence demonstrated that BNP testing is a useful tool for the diagnosis or stratification of decompensated heart failure [13,14]. Most studies have confirmed that BNP is associated with the severity of HF in a certain range. However, a study regardless of age published in 2010 demonstrated that extreme values of BNP do not correlate with the presence of HF [15]. Our results also showed that there was no significant difference in the distribution by NYHA class and LVEF in different NT pro-BNP levels. There are several possible reasons for this phenomenon. Firstly, immunoassays play an important role in falsely elevating BNP levels, which are used in the clinical setting and do not determine precise molecular forms of these natriuretic peptides. A review showed antibodies against BNP may also recognize and bind to pro-BNP and NT-fragments [16]. Meanwhile, analysis from chromatography-based studies revealed that inactive pro-BNP forms often predominate in HF,. This indicates that the bioactive forms of natriuretic peptides may not be processed proportionally in patients with advanced HF. Secondly, inflammation may be associated with falsely elevating BNP levels. Because one study had demonstrated that inflammatory markers were positively correlated with BNP levels, suggesting a link between elevations of BNP levels and inflammation [17].

Through a systematic review of 19 studies, Doust and colleagues found that NT pro-BNP is a strong prognostic indicator for both asymptomatic patients and for patients with heart failure at all stages of disease, and for every 100 ng/L rise in NT pro-BNP concentration, there was a corresponding 35% increase in the relative risk of death [18]. Additionally, a previous cohort study showed that NT pro-BNP was found to be a good predictor of mortality in elderly with and without specific cardiac diagnoses [19]. Bettencourt and colleagues [20] also demonstrated that NT pro-BNP levels were the strongest independent predictor of death or hospital readmission during hospitalization and six months of follow-up. In the extremely elevated geriatric population above 80 years, we found higher levels of NT pro-BNP with higher mortality. These results confirmed that even with the level above 3000 pg/ml and suggested that NT pro-BNP may be also an independent predictor of death in elderly patients with or without cardiac disease.

In the early phase of AMI, NT pro-BNP gene expression increases considerably, the phenomenon also can be seen in human cardiac allograft acute rejection [21]. Therefore, NT pro-BNP is a useful marker of reflection injury of myocardium function and prognostic of myocardial infarction [22]. Moreover, NT pro-BNP level is similar to BNP in circulation of healthy volunteers and it proportionally increse as cardiac function deterioration [23,24]. After compared the predictive value of NT pro-BNP and other frequently used biomarkers, Melki et al. recently found that NT pro-BNP may be the best single predictor of left ventricular function in patients with non-ST-segment elevation acute coronary syndromes [25]. In the present study, although there was no difference in the ratio of acute myocardial infarction patients in admission in different level of NT pro-BNP, we found the weak correlation between NT pro-BNP and CK-MB, and higher levels of CK-MB accompanied with higher NT pro-BNP, suggests elevated CK-MB levels derived from AMI contribute to the elevation of NT pro-BNP.

In recent years, CRP has emerged as a possible potent risk marker for cardiovascular diseases [26,27]. The correlation between CRP and BNP levels has also been demonstrated [17], which means a possible interaction between natriuretic peptides and the systemic inflammatory response. Conversely, Caroline and collegues [28] found that CRP levels are not independently associated with first cardiovascular events in older individuals, and increasing age seems to attenuate the association between plasma CRP levels and the risk of cardiovascular disease. We also observed the levels of CRP in the study, but didn’t find the correlation between NT pro-BNP and CRP.

In addition, multiple factors are known to influence the circulating levels of NT pro-BNP. The prevalence of possible influencing factors including gender, body mass index (BMI), anemia and medications for heart failure such as ACE inhibitors, angiotensin-receptor blockers, beta blockers [29-31], but in the present study, we didn’t find the correlation between BNP and all of them. Maybe these parameters are just confounding variables, and the underlying mechanism still needs to be further revealed.

There are several limitations in the present study. Firstly, the cutoff of NT pro-BNP for the enrolment and groups was established arbitrarily. Secondly, we only enrolled elderly patients above 80 years, and the population with the characteristics of more comorbidities and more severe conditions, that bring about a more confounding factor.

## Conclusions

In conclusion, our results suggest that in elderly patients above 80 years, extreme elevation of NT pro-BNP (≥3000 pg/ml) is mainly determined by impaired renal function. Extreme NT pro-BNP levels may be useful for the assessment of the severity of impaired renal function.

## Abbreviations

NT pro-BNP, N-terminal fragment pro-B-type natriuretic peptide; CK-MB, Creatine kinase MB; CKD, Chronic kidney disease; GFR, Glomerular filtration rate; CRP, C-reactive protein; HF, Heart failure; NYHA, New York heart association; CAD, Coronary artery disease; ROC, Receiver-operating characteristic; LVEF, Left ventricular ejection fraction; LVESD, Left ventricular end systolic diameter; LVEDD, Left ventricular end diastolic diameter; FS, Fractional shortening.

## Competing interests

The authors declare that they have no competing financial or any other kind of personal interests in this paper.

## Authors’ contributions

HC: obtained funding, designed the study, acquired the data, analyzed the data, interpreted of the data and drafted the manuscript. GLH: Have made substantial contributions to the design, analyses, interpretation of data and drafting the manuscript. LL: Involved in drafting the manuscript and analyzing the data. LF: Have made substantial contributions to the design, obtaining grant, analyses, interpretation of data and revising the manuscript. PY: Have made substantial contributions to the design, and analyses, interpretation of data. JC: Have made substantial contributions to the design, and analyses, interpretation of data. YYB: Have made substantial contributions to the design, analyses, interpretation of data and revising the manuscript. FW: Have made substantial contributions to the design, and analyses, interpretation of data. YXH: Have made substantial contributions to the design, and analyses, interpretation of data. All authors read and approved the final manuscript.

## Pre-publication history

The pre-publication history for this paper can be accessed here:

http://www.biomedcentral.com/1471-2261/12/57/prepub
